# Associations between meeting sleep, physical activity or screen time behaviour guidelines and academic performance in Australian school children

**DOI:** 10.1186/s12889-020-08620-w

**Published:** 2020-04-17

**Authors:** Erin K. Howie, John Joosten, Courtenay J. Harris, Leon M. Straker

**Affiliations:** 1 Department of Health, Human Performance and Recreation, University of Arkansas, Fayeteville, AR USA; 2 School of Physiotherapy and Exercise Science, Curtin University, Perth, Australia; 3 John XXIII College, Claremont, Australia; 4 School of Occupational Therapy, Social Work and Speech Pathology, Curtin University, Perth, Australia

**Keywords:** Sedentary, Education, Policy, Technology

## Abstract

**Background:**

Current guidelines suggest too little sleep, too little physical activity, and too much sedentary time are associated with poor health outcomes. These behaviours may also influence academic performance in school children. The primary purpose of this study was to examine the relationships between sleep, physical activity, or sedentary behaviours and academic performance in a school with a well-developed and integrated technology use and well-being program.

**Methods:**

This was a cross-sectional survey of students (*n* = 934, Grades 5–12) in an Australian school with a bring-your-own device (tablet or laptop computer) policy. Students reported sleep, physical activity, and sedentary (screen and non-screen) behaviours. Academic performance was obtained from school records. Linear regressions were used to test the association between behaviours and academic performance outcomes.

**Results:**

Seventy-four percent of students met sleep guidelines (9 to 11 h for children 5–13 years and 8 to 10 h for 14–17 year olds), 21% met physical activity guidelines (60 min of moderate-to-vigorous physical activity every day), and 15% met screen time guidelines (no more than 2 h recreational screen time per day); only 2% met all three. There were no associations between meeting sleep guidelines and academic performance; however later weekend bedtimes were associated with poorer academic performance (− 3.4 points on the Average Academic Index, 95%CI: − 5.0, − 1.7, *p* < .001). There were no associations between meeting physical activity guidelines and academic performance. Meeting screen guidelines was associated with higher Average Academic Index (5.8, 95%CI: 3.6, 8.0, *p* < .001), Maths 7.9, 95%CI: 4.1, 11.6, *p* < .001) and English scores (3.8, 95%CI: 1.8, 5.8, *p* < .001) and higher time in sedentary behaviours was associated with poorer academic performance, including total sedentary behaviours in hrs/day (5.8 points on Average Academic Index, 95%CI: 3.6, 8.0, *p* < .001. Meeting at least two of the three behaviour guidelines was associated with better academic performance.

**Conclusions:**

Sleep and sedentary behaviours were linked to academic performance. School communities should emphasize comprehensive wellness strategies to address multiple behaviours to maximize student health and academic success.

## Background

Throughout their day, children participate in a wide variety of behaviours including sleeping, moderate-to-vigorous physical activities such as running, and sedentary behaviours such as reading and watching television; both during and outside of school hours. Sedentary behaviour is defined as waking behaviour in a body posture that is seated or reclined, awake, and an energy expenditure less than or equal to 1.5 metabolic equivalent s[1]. Too little sleep, too little physical activity, and too much sedentary time have been associated with poor physical and mental health outcomes. These outcomes have included increased risk of obesity, impaired glucose metabolism, cardiovascular disease, depression and anxiety [2–4]. In recognition of the combined importance of sleep, physical activity and sedentary behaviours, Canada [5] and Australia have recently published 24-h ‘movement’ guidelines for children aged 5 to 17. Current guidelines are for 9 to 11 h sleep for children 5–13 years and 8 to 10 h sleep for 14–17 year olds with consistent bed and wake times, 60 min of moderate-to-vigorous physical activity per day, and no more than 2 h recreational screen time per day with limited extended sitting [5]. Perhaps most important for children, these behaviours have also been associated with poor educational outcomes [6, 7].

Poor sleep habits have been related to poor academic performance, [8] with many hypothesized mechanisms such as decreased sympathetic nervous system activity, changes in mood, inattention, decreased decision making skills, increased risk of depression, and lack of effortful control [9, 10]. However, despite the common knowledge of needing sleep to perform well in school, many questions surrounding the relationship between sleep and academic performance remain such as the importance of bedtimes, sleep duration and sleep quality [8]. For example, in a study of adolescents, self-reported sleep quality and accelerometer measured sleep duration were associated with academic performance, but not self-reported sleep duration and accelerometer measured quality of sleep, [11] highlighting a need to better understand the relationship between sleep behaviour and academic performance.

Low physical activity has been associated with poorer academic performance, [12–14] independently of the other behaviours [15]. Both acute and regular physical activity have been positively associated with improved executive functions and academic performance [12, 13], potentially resulting from a number of hypothesized mechanisms including neuroelectric changes in the brain [14], better control of glycaemic variability, [16] and an increase in brain volume and both grey and white matter density [17]. However, findings from a diverse range of research designs and outcome measures suggest physical activity is not always related to better academic outcomes [18].

Research reflects a growing interest in the relationship between sedentary behaviours and academic performance. High sedentary time may be associated with poorer academic performance through mechanisms such as decreased arousal [19]. When sedentary behaviour has been measured objectively, there have been inconsistent associations with academic performance. Lopes et al. found no association between accelerometer measured sedentary time and academic performance [20]. However, Maher et al. found that higher sedentary behaviour, measured by accelerometers, was associated with higher academic performance, [21] while Happala et al. have found higher sedentary time, measured using heart rate monitors, to be associated with poorer math and reading skills [22]. Whilst there are no current specific guidelines for overall sedentary time for children, there are time limit guidelines for leisure screen time based on prior evidence of poorer health and academic outcomes associated with this particular sedentary behaviour [23, 24]. This is in line with contemporary thinking that the context and type of sedentary behaviour is likely to be important to its association with health and academic performance [23].

**Fig. 1:**
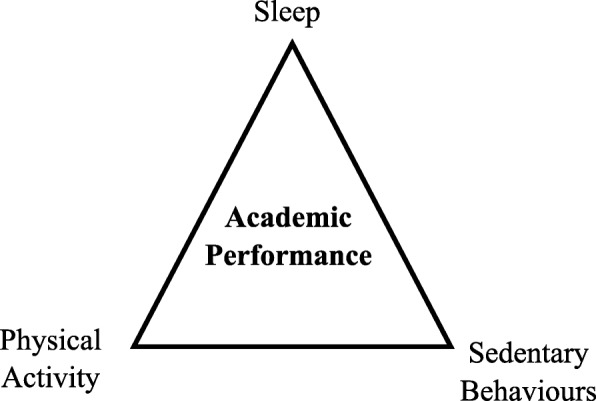
Conceptual Model of sleep, physical activity and sedentary behaviours interacting with each other and academic performance

Little research has attempted to examine the associations of academic performance with multiple behaviours, as proposed in Fig. 1, and thus be consistent with emerging recommendations. A recent cluster analysis of sleep, physical activity, sedentary behaviour, screen time and diet found that certain behaviour patterns, particularly in children who had unhealthy diets and high amounts of screen time, were related to poorer academic performance [7]. However, no studies have examined these three behaviours across school grades or used school-specific academic performance outcomes, which may precede national standardized test score results, as an earlier and more specific indicator of poor academic performance.

Thus, the primary aim of this study was to examine the individual relationships between meeting sleep, physical activity, or sedentary behaviour guidelines and academic performance in a school with a well-developed and integrated technology use and well-being program. Secondary aims included examining whether the three behaviours were related to each other, whether meeting at least 2 behaviour guidelines was associated with academic performance, and whether other measures of these behaviours were associated with academic performance. It was hypothesized that meeting sleep, physical activity or sedentary behaviour guidelines, or at least 2 of the 3 guidelines, would be associated with higher academic performance. It was also hypothesized that the 3 behaviours would be related to each other and that longer sleep, higher physical activity and lower sedentary time would be associated with higher academic performance. Understanding these relationships is critical for schools to develop policies to maximize health and educational outcomes for their students.

## Methods

### Study design

The current study was a cross-sectional survey of students. A sub-sample of students repeated the survey for test-retest reliability assessment.

### Participants

Participants were a convenience sample of children and adolescents attending an independent, socially-advantaged school in Perth, Western Australia (total of approximately 1470 students). According to the Index of Community Socio-Educational Advantage (ICSEA), the selected school was in the top 6% of schools nationally based on student academic performance, parent education and income, school remoteness, and proportion of Indigenous students. Since 2012, the school has moved through several iterations of a 1-to-1 device program for its students where each student has access to a computer device. For the year of data collection, students in Grades 5 through 9 were required to “bring-your-own” iPad (supplied by individual students). Students in Grades 10 through 12 had mandatory bring-your-own laptops or tablets. Parents received an announcement of the study in a school letter with an opportunity to withdraw consent prior to their child completing the survey. No parents requested their children not participate. All children in Grades 5 through 12 were invited to participate through their homeroom classrooms as per principal request and school approval. Children provided informed assent electronically before beginning the survey. This study was approved by the Curtin University Human Research Ethics Committee (Approval Number 174–15).

### Procedure

The survey was conducted during August 2016 and administered online through Qualtrics (see Appendix 1 for a complete copy of the questionnaire). Children completed the electronic survey during a homeroom class, with a research staff member present to answer questions. Hard copy surveys were available upon request but none were requested. No complete names or further identifying information were collected and once the data were linked between multiple surveys (for reliability assessment) and to academic performance, the student IDs were removed and replaced by a unique study participant ID by the school Director of Information and Communications Technology. A subsample of students, one classroom from each of Grades 5, 7, 8, 10 and 11, completed the survey again two-weeks after the original completion.

### Instrumentation

*Sleep*. The amount and quality of sleep were obtained from questions selected from the Children’s Report of Sleep Patterns [25]. These included two multiple choice questions about the quality of sleep and amount of sleep. Four additional questions were included on usual weekday and weekend bedtimes and wake times with multiple-choice selections in half hour increments (e.g. 7:30–7:59 pm, 8:00–8:29 pm). Meeting the sleep behaviour guidelines was characterized as 9 to 11 h for children 5–13 years and 8 to 10 h for 14–17 year olds. Other sleep behaviour variables considered in secondary analysis were hours of sleep on weekdays and weekend days, weekday and weekday bedtimes later than 11 pm and self-reported sleep quality.

#### Physical activity

Physical activity was reported through questions designed in congruence with the Australian National Physical Activity Report Card working group and based on previous questionnaires [26, 27]. These questions were designed to capture total physical activity, organized team and individual sports and non-organized physical activity. The primary physical activity question was “Thinking about the last 7 days, on how many days did you do physical activity for at least 60 minutes that increased your heart rate so you were out of breath? The 60 minutes each day could be broken up in shorter bursts throughout the day.” A range of answer selections from “no days” to “7 days” were given. Meeting the physical activity guideline was characterized as 60 min of moderate-to-vigorous physical activity (MVPA) every day. Other physical activity variables considered for secondary analysis were days getting 60 min of MVPA, participating in sports and hours of unorganized play per day.

#### Sedentary behaviours

Time spent in sedentary behaviours was self-reported by children via questions on screen technology use by device and purpose of use and non-screen activities including homework/school work (not on computer), reading books (not on computer), playing musical instrument, arts & crafts, other (e.g. socializing) using the Technology Use Questionnaire (TechU-Q) [28]. Reliability was moderate to high for individual items and has been published elsewhere [28]. Frequency and duration of use were used to calculate average daily use. Reports of greater than 12 h a day exposure for any individual sedentary behaviour were considered implausible and excluded from analysis. To assess if students were meeting available sedentary guidelines, a leisure screen behaviours variable was created by summing TV, electronic games (handheld, console, active), and non-educational use reported for desktop, laptop, tablet computers and mobile phones. Reports of greater than 3600 min were excluded as unrealistic responses. To create a total sedentary variable, educational activities on the desktop, laptop, tablet computers and mobile phones computers along with the additional non-screen sedentary activities, were added to the leisure screen use variable. The summed variables did not account for potential multitasking and thus could exceed daily waking hours. Meeting the screen time behaviour guidelines was characterized as no more than 2 h recreational screen time per day. Other sedentary behaviour variables considered in secondary analysis were total hours of leisure screen behaviours per day and total hours of sedentary behaviors per day.

#### Academic performance

School class, subjects, and subject scores were used to develop an academic index, in consultation with senior teachers and used by the school for internal evaluations as an alternative to Grade Point Average. For Grades 5–6, subject grades (A, B, C, D) were assigned a score of 90, 80, 70, 60 respectively. The Average Academic Index for Grades 5 and 6 included the average score for all 11 available core subjects. Individual subject scores for Maths and English were also used in further analysis. For Grades 7 through 10, the Average Academic Index included an average of final exam scores for core subjects Maths, Science, English, Humanities and Religion (elective subjects such as Drama Skills or Philosophy, not taken by all students, were excluded). Subjects for which there were multiple levels which varied by grade (to cater for different levels of academic ability) were weighted based on instructor consensus. The average score was calculated for three separate academic scores: Average Academic Index and individual subject scores for Maths and English. Academic scores could range from 0 to 100 with 100 being a high academic performance.

Additional demographic information including date of birth or gender were reported by students.

### Data analysis

Descriptive statistics were calculated with means and standard deviations presented for the variables used to characterise each behaviour. Sedentary and leisure screen use variables were non-normally distributed (as determined by visual inspection of histograms), and median and 25th to 75th percentiles were examined and displayed the same pattern, thus means are presented in tables for consistency with medians presented in the text. Behaviours were compared using Chi-square for categorical variables, t-tests for normally distributed variables and Wilcoxan rank sum for non-normally distributed variables. Intraclass correlations (ICC) between the two surveys completed by a sub-sample were calculated for sleep, physical activity, and sedentary behaviour variables. To test the associations among sleep, physical activity and sedentary behaviours, linear regression, logistic regression, ordinal logistic regression were used depending on the dependent variable. Regressions were used to test the associations between meeting at least 2 out of 3 guidelines, and the dependent variables of academic performance. Similarly, regression models were used to test the associations between the other measures of sleep, physical activity and sedentary behaviours and academic performance. All models were adjusted for gender and school grade and the distribution of residuals were checked for normal distribution to ensure appropriate model fit.

## Results

A total of 978 children began the survey. Forty-four children did not provide valid ID’s or identifying information and were not included in the analyses. This resulted in a total available sample of 934 children (50% girls, mean age 14.7 (SD 2.7). Six children did not answer any of the technology questions and 96 children did not answer any of the non-screen sedentary behaviour questions. Total daily technology use greater than 3600 min per day (5 or more devices with max use of 12 h per day), purpose of screen use exceeding 60 h for any device (5 purposes with a max of 12 h per day) and total of non-screen sedentary activities greater than 3600 min per day (5 activities with max of 12 h per day) were considered to be implausible and those data were excluded for sedentary behaviour responses. All remaining data were used in relevant analyses. As not all children completed each question, a description of varying sample sizes, as well as a description of behaviours, can be seen in Table 1.

**Table 1:** Behaviours reported by school children by gender mean (SD) or n (%)

		Total n	Total	Girls	Boys	***p***-value^**a**^
**Sleep**	% meeting sleep guidelines ^c^	828	611 (74%)	307 (74%)	304 (73%)	.724
	Weekday sleep (hrs/night) ^b^	830	9.0 (1.2)	9.1 (1.1)	9.0 (1.3)	.525
	Weekend sleep (hrs/night) ^b^	828	9.7 (1.4)	9.8 (1.2)	9.6 (1.5)	.058
	Weekday bedtime ≥23:00	830	101 (12%)	51 (12%)	50 (12%)	.915
	Weekend bedtime ≥23:00	829	258 (31%)	138 (33%)	120 (29%)	.185
	% poor sleeper	829	83 (10%)	30 (7%)	53 (13%)	.052
**Physical Activity**	% meeting guidelines ^d^	840	174 (21%)	60 (14%)	114 (27%)	<.001
	Days getting 60 min MVPA	840	4.3 (2.1)	4.0 (2.0)	4.6 (2.2)	<.001
	Unorganized play (hrs/day)	837	1.7 (2.0)	1.5 (1.8)	1.9 (2.3)	<.001
	% participating in sport	838	769 (92%)	380 (91%)	389 (93%)	.258
**Sedentary Behaviours**	% meeting screen time guidelines ^e^	832	95 (11%)	59 (14%)	36 (9%)	.018
	Leisure screen behaviours (hrs/day)	832	12.1 (15.4)	10.0 (11.1)	14.2(18.5)	<.001
	Total sedentary behaviours (hrs/day)	832	21.4 (19.8)	19.9 (15.1)	23.0 (23.6)	.395
**Academic scores**	Average Academic Index	933	67.1 (11.3)	68.6 (10.8)	65.6 (11.5)	<.001
	Maths	925	58.3 (19.7)	57.0 (19.0)	59.5 (20.1)	.057
	English	864	70.0 (9.8)	73.0 (9.1)	67.3 (9.6)	<.001
**% meeting all 3 guidelines**		824	17 (2%)	6 (1%)	11 (3%)	.217
**% meeting at least 2/3 guidelines**		824	188 (23%)	84 (20%)	104 (25%)	.039

^a^T test, wilcoxan rank, or chi-squared test for gender differences

^b^Sleep time from reported usual bedtimes and waketimes

^c^Sleep guidelines 9 to 11 h for children 5–13 years and 8 to 10 h for 14–17 year olds

^d^60 min of physical activity on 7 days per week

^e^screen time guidelines of less than 2 h of leisure screen activity

### Reliability

The ICC for reported typical sleep duration per night was .86 (95%CI: .78, .89) for weekdays and .57 (.42, .69) on weekends. The ICC for days of meeting physical activity guidelines was .70 (.59, .79) and .25 (.06, .42) for unorganized play. The ICC for leisure screen behaviours was .89 (.84, .93) and .86 (.79, .90) for total sedentary behaviours.

### Sleep

A total of 74% of children met age-appropriate sleep guidelines (see Table 1), ranging from 78% of Grade 5 students to 70% of Grade 12 students (see Supplemental Table A). The mean hours of sleep per night was 9.0 (SD 1.2) on a weekday and 9.7 (1.4) on a weekend and did not differ between girls and boys. This ranged from 9.9 (1.2) hours on a weekday for Grade 5 students to 8.3 (1.1) hours in Grade 12 students and 9.6 (1.5) hours on a weekend for Grade 5 students to 9.2 (1.1) hours in Grade 12 students (see Supplemental Table A). On weekdays, 12% of children reported going to bed at 11 pm or later, while 31% reported going to bed past 11 pm on weekends. While 25% of Grade 5 students reported getting too little sleep, 66% of Grade 12 students reported too little sleep. Ten percent of students reported poor sleep, with little variation between Grades.

### Physical activity

Twenty-one percent of children reported meeting physical activity guidelines of 60 min on 7 days per week. Children reported achieving at least 60 min of physical activity on an average of 4.3 (SD 2.1) days per week (see Table 1). Fourteen percent of girls reported meeting physical activity guidelines, compared to 27% of boys. When examined by school Grade, 44% of Grade 5 students reported meeting these guidelines and this decreased to only 9% in Grade 12 (see Supplemental Table A). 92% of children reported participating in a sport, and on average reported 1.7 (SD 2.0) hours per day of unorganized play.

### Sedentary Behaviours

Only 11% of students met screen guidelines and there were no statistical differences between girls and boys (see Table 1). Students reported leisure screen use of 12.1 (SD 15.4) mean hours per day (median 7.4 h per day; 25th percentile 3.9, 75th percentile 13.6). Leisure screen use was lower (*p* < .001) for girls (10.0 (SD 11.1) hours per day, median 6.4) than boys (14.2 (SD 18.5) hours per day, median 8.7). Students reported total sedentary behaviours of 21.4 (SD 19.8) mean hours a day (median 16.0 h; 25th percentile 10.5, 75th percentile 24.5). There was not a statistically significant difference between girls and boys as seen in Table 1. Total sedentary behaviour time ranged from 13.7 h per day in Grade 5 to 18.8 h in Grade 12.

### Academic performance

The mean Average Academic Index, Maths, and English Scores are seen in Table 1 and broken down by school grade in Supplemental Table B.

### Associations between sleep, physical activity and sedentary behaviours

Only 2% of children met all three behaviour guidelines, with 23% meeting at least 2 out of 3 guidelines (Table 1).

#### Sleep & physical activity

Children who met sleep guidelines were less likely to have achieved 60 min of more of physical activity each day (OR 0.71, 95%CI: 0.54, 0.94, *p* = .016). Children who reported meeting physical activity guidelines reported 0.29 (0.10, 0.48, *p* = .002) hours less sleep on weekdays and 0.27 (0.03, 0.50, *p* = .026) hours less sleep on weekends. Each additional day of achieving 60 min of physical activity was associated with fewer hours of sleep on weekdays (unstandardized β − 0.04, 95%CI: − 0.08, − 0.00002, *p* = .049) but not on weekends. There were no differences in sleep by reported sport participation.

#### Sleep & sedentary

Children who met sleep guidelines reported less time in sedentary behaviours (− 6.0 h per day, 95%CI: − 9.1, − 2.9, *p* < .001) and leisure screen behaviours (− 4.8, 95%CI: 07.2, − 2.4, *p* < .001). Children who met leisure screen guidelines reported more weekday (0.46 h per night, 95%CI: 0.23, 0.70, *p* < .001) and weekend sleep (0.35, 95%CI: 0.05, 0.64, *p* = .022). Children who reported going to bed after 11 pm on weekdays (8.6 h, 95%CI: 5.2, 12.0) and weekends (7.2, 95%CI: 4.8, 9.6) reported higher leisure screen behaviours than those who went to bed before 11 pm. Hours reported in leisure screen behaviours were negatively associated with hours of sleep on weekdays (− 0.01, 95%CI: − 0.02, − 0.01, *p* < .001) but not weekend days. Similarly, total hours of sedentary behaviours were negatively associated with hours of sleep on the weekdays (− 0.01 h, 95%CI: − 0.01, − 0.006, *p* < .001) but not on weekends.

#### Sedentary & physical activity

There were no associations between meeting physical activity guidelines and leisure screen behaviours or total hours of sedentary behaviours. Nor were there no associations between meeting leisure screen guidelines and days of participating in 60 min of physical activity.

### Associations between Behaviours and academic performance

There was a positive association between meeting at least 2 out of 3 guidelines and Average Academic Index, Maths and English as seen in Table 2.

**Table 2:** Associations between 3 behaviours and Average Academic Index, Maths and English academic performance

	Average Academic Index			Maths			English		
	N	Β (95%CI)	*P*-value	N	β	*P*-value	N	β	*P*-value
**Sleep**									
Weekday sleep (hrs/night)	829	0.05 (− 0.6, 0.7)	.888	824	−0.3 (−1.4, 0.8)	.614	772	0.02 (−0.6, 0.6)	.954
Weekend sleep (hrs/night)	827	0.3 (− 0.2, 0.8)	.241	822	0.03 (− 0.9, 0.9)	.955	770	**0.5 (0.06, 1.0)**	**.028**
Weekday bedtime ≥23:00	829	−1.1 (−3.3, 1.2)	.365	824	0.2 (−3.7, 4.0)	.939	772	−1.3 (− 3.4, 0.9)	.245
Weekend bedtime ≥23:00	828	**−3.4 (−5.0, −1.7)**	**<.001**	823	**−3.9 (−6.7, − 1.1)**	**.006**	771	**−2.1 (− 3.6, −0.6)**	**.005**
Poor sleep quality (vs not poor)	813	1.3 (−1.2, 3.8)	.305	808	**1.3 (− 2.9, 5.5)**	**.549**	756	**−0.1 (−2.3, 2.2)**	**.958**
Meeting sleep guidelines	827	1.1 (−0.5, 2.8)	.170	822	0.7 (−2.1, 3.5)	.621	770	1.0 (−0.5, 2.5)	.174
**PA**									
Days getting 60 min MVPA	839	0.1 (−0.3, 0.5)	.586	834	0.2 (−0.4, 0.8)	.561	782	0.1 (−0.2, 0.4)	.632
Participated in sport	837	0.3 (−2.3, 2.8)	.847	832	0.7 (−3.7, 5.1)	.747	780	−0.1 (−2.5, 2.3)	.916
Unorganized play (hr/day)	836	**−1.0 (−1.3, −0.6)**	**<.001**	831	**− 1.5 (−2.1, − 0.9)**	**<.001**	779	**−0.7 (− 1.0, − 0.3)**	**<.001**
Meeting PA guidelines	839	0.3 (− 1.5, 2.1)	.784	834	1.2 (− 1.9, 4.3)	.442	782	0.1 (− 1.5, 1.7)	.883
**Sedentary**									
Leisure screen behaviours (hrs/day)	831	**−0.2 (− 0.3, − 0.2)**	**<.001**	826	**−0.3 (− 0.4, − 0.3)**	**<.001**	772	**−0.1 (− 0.2, − 0.1)**	**<.001**
Total sedentary behaviours (hrs/day)	831	**− 0.1 (− 0.2, − 0.1)**	**<.001**	826	**−0.2 (− 0.3, − 0.2)**	**<.001**	772	**−0.1 (− 0.1, − 0.06)**	**<.001**
Meeting screen guidelines	831	**5.8 (3.6, 8.0)**	**<.001**	826	**7.9 (4.1, 11.6)**	**<.001**	772	**3.8 (1.8, 5.8)**	**<.001**
**Meeting at least 2/3 guidelines (%)**	**833**	**2.3 (0.5, 4.0)**	**.010**	**828**	**3.4 (0.5, 6.4)**	**.021**	**774**	**1.6 (0.05, 3.1)**	**.043**

^a^ General use e.g. visiting websites, online shopping, downloading music

#### Sleep & academic performance

There were no associations between meeting sleep guidelines and academic performance (see Table 2). Hours of sleep on weekends were positively associated with English scores (β 0.5, 95%CI:, 0.06, 1.0, *p* = .028) as seen in Table 2. Hours of sleep on weekdays and weekends were not associated with Average Academic Index or Maths. Later weekend bedtimes were associated with poorer Average Academic Index (− 3.4, 95%CI: − 5.0, − 1.7, *p* < .001), Maths (− 3.9, 95%CI: − 6.7, − 1.1, *p* < .001), and English (− 2.1, 95%CI: − 3.6, − 0.6, *p* = .001) scores. However, there were no associations between weekday bedtimes and academic performance.

#### Physical activity & academic performance

There were no associations between days of meeting physical activity guidelines and academic performance (Table 2). Participating in a sport was not associated with academic performance although hours reported in unorganized play was negatively associated with Average Academic Index (− 1.0, 95%CI: − 1.3, − 0.6, *p* < .001), Maths (− 1.5, 95%CI: − 2.1, − 0.9, *p* < .001), and English (− 0.7, 95%CI: − 1.0, − 0.3, *p* < .001) scores.

#### Sedentary & academic performance

Meeting screen guidelines was associated with higher Average Academic Index (5.8, 95%CI: 3.6, 8.0, *p* < .001), Maths 7.9, 95%CI: 4.1, 11.6, *p* < .001) and English scores (3.8, 95%CI: 1.8, 5.8, *p* < .001) (Table 2). Hours of leisure screen behaviours and total sedentary behaviours were negatively associated with the Average Academic Index, Maths and English scores.

## Discussion

Overall, almost no students in this sample from an advantaged school met all three guidelines for sleep, physical activity and sedentary behaviours with a majority not meeting screen time or physical activity guidelines.

Of the three behaviours, the highest percentage (70%) of students were meeting sleep guidelines. There were no differences in amount of sleep reported by girls and boys; however, less sleep and later bedtimes were reported by older children [8]. Additionally, more older children reported getting too little sleep. These results suggest school communities (parents and schools) need to work together to maintain positive sleep habits as children get older into adolescence and beyond.

Unfortunately, only 1 in 5 students met current physical activity guidelines, despite the school focusing on overall health and wellness. Boys and younger children reported more physical activity than girls and older children, which is consistent with previously reported data [29]. The low percentage of children meeting physical activity guidelines is despite 90% of children reporting participating in sport in the past year. This supports the recent conclusion from the 2014 Australian National Physical Activity Report Card, that sport may not be enough for ensuring children meet physical activity guidelines for health [30]. This may be due to specialization or training schedules that do not meet every day, or all year, and children not participating in other physical activity opportunities. Initiatives other than sports are required to encourage physical activity in a school context and build on current school programs. These could include enhanced promotion of active lifestyles in physical education, effective supports for activity between classes and before and after school, and use of activity during classes.

There are no current specific guidelines for total sedentary behaviours, but adult and child public health guidelines recommend minimizing sedentary time and reducing prolonged periods by breaking it up into shorter bouts of sedentary time [31]. Children in the current study reported a mean of over 20 h per day of sedentary behaviours, not including school lessons. This is likely to be an overestimate of absolute time due to known inaccuracies with self-report and also as it was derived as a sum of multiple purposes of use (and thus does not account for multitasking). This suggests that there is a high amount of multitasking occurring, and a single item may not capture all the purposes of use which are likely to have unique effects on health and development. For example, children may be working on homework while socializing with friends on their mobile phone. It is indicative of a high amount of screen and sedentary behaviours, and thus the need to reduce and potentially break up sedentary times. Strategies, such as replacing some sitting time with standing, activity breaks in lessons or more active learning, can help to break up existing periods.

Leisure screen use made up a large percentage of total sedentary behaviours for this sample. Students in the current study reported over 12 h per day of leisure screen use, which was over half of total sedentary behaviour time. When trying to reduce sedentary behaviours of school children, technology use may be a key point of intervention. However, students still participate in a large amount of non-screen behaviours, making it important to distinguish between screen behaviours and sedentary behaviours and not use them as proxies for each other.

Current guidelines for Australian children are to limit the use of screens for entertainment to less than 2 h per day [31]. Overall, only 11% of students met this guideline with most students reporting over 12 h per day using technology for purposes other than school work. This did not account for multitasking that likely occurs, however, it is still remarkably higher than the recommended 2 hours. A previous study of Australian children aged 8 to 16 years found 55% of 8-year-olds met the screen time guidelines while only 20% of 16 year olds did [32]. One simple suggestion for school communities is to communicate to parents to decrease leisure screen use may be to limit children’s bedroom access and use of screens, as bedroom access and use have been related to poorer outcomes in previous studies [33, 34].

The current public health sedentary behaviour guidelines need to be reconsidered in light of the ubiquity of screen based devices and the increased educational use of technology. As educational and non-educational use likely has the same physiological and health effects, revised public health guidelines should provide recommendations for overall sedentary time limits. Specific guidance will also be needed on particular sedentary behaviours, including non-screen behaviours, screen-based school related behaviours and screen-based leisure behaviours, derived from a better understanding of how these relate to health and educational outcomes.

There were several associations between behaviours. More time spent in sedentary behaviours was associated with less sleep. Specifically, higher technology use was associated with less sleep and later bedtimes, suggesting previous conclusions that the effect of technology use on less sleep may be through later bedtimes [35]. Contrary to hypotheses, higher physical activity was associated with *less* sleep on weekdays. This may be the result of early awakening for before-school training sessions or time spent in sport or physical activity later in the day being in addition to time for homework resulting in later bedtimes. This is important for school communities to consider as policies that change one behaviour may effect another (e.g. increasing physical activity may result in later bedtimes), or that behaviours may be indicative of overall behavioural profiles (e.g. social individuals with high sedentary and high physical activity) [36].

Importantly, these behaviours were associated with academic performance. Most consistently, higher total sedentary behaviours, including leisure screen use, were negatively associated with all academic performance outcomes in this study. While the effects were small, meeting screen guidelines was associated with 2 to 5 percentage points higher on the academic index. This may make a difference in tertiary options available to a student in an increasingly competitive society. Combined with other factors, such as late weekend bedtimes, these behaviours may lead to poorer academic outcomes, which could be prevented through comprehensive health strategies [37]. Schools need advice on how to support children and their families to reduce total sedentary time, for health benefits, whilst enabling sufficient sedentary time to succeed with academic development.

There were minimal associations between sleep or physical activity and academic performance in the current study. It is possible that sleep on weekends is more variable and dependent on the child, while weekday bedtimes are more structured and may be more regulated by parents. There was no association between meeting physical activity guidelines and academic performance in the current study, however higher unorganized play was associated with poorer academic performance. This could be a reflection of poorer time management with children playing instead of doing homework, or the result of other confounders such as socioeconomic status with children of higher socioeconomic status involved in more organized play. Previous research suggests a positive association between physical activity and academic performance, [38] however, the physical activity measures used in this study were broad, and did include detailed information on different types of physical activity. As with sedentary behaviours, different types of physical activity may have different associations with academic performance. Additionally, activities like sports participation were high in this population, so it was not surprising that no relationships were found between academic performance and sports participation due to little variation within the sample.

Whilst meeting individual sleep or physical activity guidelines was not clearly related to better academic performance, meeting at least 2 out of the 3 guidelines was associated with improved academic performance. Prior research has found similar associations of meeting multiple guidelines with better physical health outcomes [39, 40] as well as impulsivity [41] and general cognitive function [42]. Earlier studies have also shown that health related behaviours often cluster together in children [43, 44]. This provides support for schools to encourage more positive habits across all three behaviours. There is ongoing debate about whether interventions are more effective when targeted towards a single behaviour (for example physical activity) rather than multiple behaviours (for example physical activity, sedentary behaviour and sleep) [7, 45, 46]. A single behaviour target may be more effective in changing a single behaviour, but a strategy targeting multiple behaviours may be more effective in enhancing an outcome that is influenced by multiple behaviours, such as obesity [46] or academic performance. Further research could evaluate the most effective strategies for enhancing academic performance, although health outcomes are likely to always be considered important by society.

One limitation of this study was the use of self-reported technology use, however, there are limited objective measures of technology use (e.g. direct observation) that can be used in a large sample. Similarly, sleep and physical activity were self-reported. Objective measures such as accelerometers and device monitoring software (i.e. app tracking software) may provide more accurate total length of time, but miss contextual information of multi-tasking and are limited to a device such as tracking on a mobile phone rather than all technology devices. However, the measures used had moderate to high reliability, except for the unorganized play item, which may be a highly variable behaviour (e.g. weather dependent). Further scattergrams supported the associations findings, suggesting self reports may be adequate relative exposures. Academic performance was not self-reported but obtained directly from school records, reducing bias from student reporting their own performance. Only one homogenous school was used with no adjustment for individual variation in socioeconomic status, which limited generalizability but increased internal validity by minimising variation in potential several confounders such as socioeconomic status. Other factors such as student body mass index, which may be moderators of the relationship, were not assessed.

## Conclusions

Few students in this study met sleep, physical activity and screen guidelines. There was high overall time in sedentary behaviours and leisure screen use reported by students in a school with a well-developed technology policy. Higher technology use was associated with poorer academic performance in the current study, as was failing to meet at least 2 of the 3 behaviour guidelines. School communities have a role to play in ensuring not only the academic success, but also the health and wellness of students and establishing healthy behaviours for lifelong habits. As these behaviours are also linked to academic performance, the need to establish, implement, and evaluate comprehensive wellness policies and strategies to address multiple behaviours should be a priority for school administrators, teachers and their school communities.

### Practical implications

Schools and school communities requiring technology for educational purposes should make efforts to reduce the total time spent on sedentary behaviours. These strategies may include limiting bedroom use of technology, parent monitoring of devices, co-participation, or role modeling. To minimize displacement of beneficial activities, schools should offer and promote physical activity opportunities in addition to sports including non-competitive afterschool activities and lifelong, lifestyle activities (i.e. jogging, dance, fitness). As behaviours are related, wellness policies should be comprehensive and include policies and strategies for positive sleep, physical activity, and sedentary behaviours.

## Supplementary information

**Additional file 1: Supplemental Table A**: Activity behaviours reported by school children by school Grade. **Supplemental Table B:** Academic performance by school Grade, mean (SD).

**Additional file 2.** Technology Use Questionnaire (TechU-Q).

## Data Availability

The data that support the findings of this study are available by request from the authors.

## Abbreviations

ICSEA: Index of Community Socio-Educational Advantage
MVPA: moderate-to-vigorous physical activity
TechU-Q: Technology Use Questionnaire
ICC: Intraclass correlations
