# Supplementary material for: Associations between meeting sleep, physical activity or screen time behaviour guidelines and academic performance in Australian school children
Source: BMC Public Health. 2020 Apr 17;20:520. doi: 10.1186/s12889-020-08620-w (PMC7165394; doi:10.1186/s12889-020-08620-w)
Supplement: Supplementary file 1 — Additional file 1: Supplemental Table A: Activity behaviours reported by school children by school Grade. Supplemental Table B: Academic performance by school Grade, mean (SD). [file 12889_2020_8620_MOESM1_ESM.docx]

**Supplemental Table A**: Activity behaviours reported by school children by school Grade

|  | **Grade 5** | **Grade 6** | **Grade 7** | **Grade 8** | **Grade 9** | **Grade 10** | **Grade 11** | **Grade 12** |
| --- | --- | --- | --- | --- | --- | --- | --- | --- |
| **Sleep (n^a^)** | 51 | 54 | 173 | 152 | 144 | 89 | 97 | 70 |
| Weekday sleep (hrs) | 9.9 (1.2) | 9.8 (1.0) | 9.6 (1.0) | 9.2 (1.1) | 8.8 (1.2) | 8.7 (0.9) | 8.2 (1.2) | 8.3 (1.1) |
| Weekend sleep (hrs) | 9.6 (1.5) | 10.0 (1.4) | 10.0 (1.3) | 9.8 (1.4) | 9.5 (1.4) | 9.6 (1.5) | 9.3 (1.3) | 9.2 (1.1) |
| Weekday bedtime >23:00 | 1 (2%) | 1 (2%) | 4 (2%) | 13 (9%) | 22 (15%) | 10 (11%) | 26 (27%) | 24 (34%) |
| Weekend bedtime >23:00 | 5 (10%) | 4 (7%) | 22 (13%) | 35 (23%) | 64 (44%) | 34 (38%) | 52 (54%) | 42 (61%) |
| % meeting sleep guidelines | 39 (78%) | 42 (78%) | 132 (76%) | 109 (72%) | 109 (76%) | 72 (81%) | 60 (62%) | 48 (70%) |
| How much sleep? *Too much* | 4 (8%) | 1 (2%) | 3 (2%) | 8 (5%) | 2 (1%) | 2 (2%) | 4 (4%) | 2 (3%) |
| *Too little* | 13 (25%) | 13 (24%) | 48 (28%) | 45 (30%) | 64 (44%) | 45 (51%) | 59 (61%) | 46 (66%) |
| Poor sleeper | 7 (13%) | 5 (9%) | 13 (8%) | 17 (11%) | 11 (8%) | 12 (13%) | 10 (10%) | 8 (11%) |
| **Physical Activity (n^b^)** | 52 | 54 | 173 | 152 | 147 | 89 | 99 | 74 |
| Days getting 60 min MVPA | 5.2 (2.1) | 4.9 (1.9) | 4.7 (1.9) | 4.6 (1.9) | 4.2 (2.1) | 4.1 (2.1) | 3.4 (2.1) | 2.9 (2.2) |
| Participated in sport | 49 (94%) | 53 (98%) | 160 (92%) | 139 (91%) | 139 (95%) | 76 (85%) | 85 (87%) | 68 (93%) |
| Participated in team sport | 48 (92%) | 53 (98%) | 155 (90%) | 130 (86%) | 131 (89%) | 68 (76%) | 72 (72%) | 52 (71%) |
| Participated in individual sport | 17 (33%) | 20 (37%) | 52 (30%) | 55 (36%) | 57 (39%) | 30 (34%) | 39 (40%) | 40 (55%) |
| Unorganized play (hr/day) | 1.3 (1.1) | 2.0 (2.2) | 1.9 (2.2) | 1.9 (2.2) | 1.8 (2.2) | 1.6 (2.1) | 1.3 (2.1) | 1.0 (1.2) |
| Meeting PA guidelines | 23 (44%) | 18 (33%) | 40 (23%) | 33 (22%) | 29 (20%) | 15 (17%) | 9 (9%) | 7 (9%) |
| **Sedentary (n)** | 52 | 53 | 171 | 149 | 145 | 90 | 99 | 73 |
| Leisure screen behaviours | 5.7 (7.0) | 6.9 (9.0) | 9.7 (10.3) | 14.2 (17.1) | 16.9 (21.6) | 14.7 (18.1) | 12.3 (12.9) | 8.6 (8.7) |
| Total sedentary behaviours | 13.7 (9.9) | 14.5 (10.9) | 18.5 (12.5) | 22.2 (20.5) | 26.3 (28.1) | 25.3 (24.3) | 24.5 (19.4) | 18.8 (11.9) |

^a^ n for weekday sleep

^b^ n for Days getting 60 min MVPA

**Supplemental Table B: Academic performance by school Grade, mean (SD)**

|  | **Grade 5**  **(n** ^a^=52) | **Grade 6**  **(n=53)** | **Grade 7**  **(n=173)** | **Grade 8**  **(n=160)** | **Grade 9**  **(n=160)** | **Grade 10**  **(n=111)** | **Grade 11**  **(n=110)** | **Grade 12**  **(n=109)** |
| --- | --- | --- | --- | --- | --- | --- | --- | --- |
| Average Academic Index | 77.2 (4.4) | 78.2 (5.0) | 71.2 (11.0) | 65.5 (11.4) | 64.6 (11.7) | 64.6 (11.6) | 62.2 (8.6) | 63.8 (8.9) |
| Maths | 79.6 (7.9) | 79.8 (7.2) | 70.1 (16.2) | 50.6 (19.5) | 53.3 (19.8) | 53.8 (18.0) | 49.5 (13.2) | 50.3 (14.7) |
| English | 76.0 (7.2) | 76.8 (7.5) | 71.6 (11.1) | 69.4 (9.7) | 69.5 (9.2) | 70.4 (9.6) | 64.0 (7.0) | 65.8 (7.5) |

^a^ n for Core
