# Supplementary material for: Associations between meeting sleep, physical activity or screen time behaviour guidelines and academic performance in Australian school children
Source: BMC Public Health. 2020 Apr 17;20:520. doi: 10.1186/s12889-020-08620-w (PMC7165394; doi:10.1186/s12889-020-08620-w)
Supplement: Supplementary file 2 — Additional file 2. Technology Use Questionnaire (TechU-Q). [file 12889_2020_8620_MOESM2_ESM.pdf]

JTC TechU-Q - final

***the Qualtrics version of the survey with images can be obtained by contacting the authors at ekhowie@uark.edu**

Thank you for taking the time to fill in this survey.

This survey is trying to find out how teenagers are using touch screen devices, such as tablet computers and smart phones today. We will ask you questions on your use of various electronic devices, and some aspects of your life that might be influenced by your use of devices. By taking part in this survey, you will be providing valuable new information to help guide wise use of these devices.

By completing the survey, you give assent to participate in this study, and agree that you have been informed of and understand the purpose of this study. Participation or non-participation in this study will have no effect on your grades or performance in school. Information provided by you will be kept confidential. This study has been approved by the Curtin University Human Research Ethics committee (RDHS-100-15).

Please read each question carefully and answer ALL of the questions.

(fields marked with * are required)

○ ***What is your Student ID? (use your network login code)**

____

Click on the arrow below to start the survey.
Please read each question carefully and answer ALL of the questions.

Some questions about you…

1. *When were you born?

	Month	Day	Year
Please select:	▼ January ... December	▼ 1 ... 31	▼ 1900 ... 2049

2. *What year are you in at school now?

- ○ 5
- ○ 6
- ○ 7
- ○ 8
- ○ 9
- ○ 10
- ○ 11
- ○ 12

What is your height and weight? (enter "don't know" if unsure)

- ○ Height (cm) ____
- ○ Weight (kg) ____

4. *Are you a boy or girl?

- ○ Boy
- ○ Girl

***What devices do YOU use, which do YOU own (have for yourself or bought with your own money), and use in your bedroom?**

	Do you **personally have or own** any of the following devices?		Do you use these in your **bedroom**?		Have you used any of these devices in the **past 12 months**?	
	Yes	No	Yes	No	Yes	No

**Television**	○	○	○	○	○	○
**Desktop computer**	○	○	○	○	○	○
**Laptop computer**	○	○	○	○	○	○
**Tablet computer** (e.g. iPad, Samsung Galaxy Tab, Kindle e-reader, Microsoft Surface Pro)	○	○	○	○	○	○
**Mobile phone - <u>WITH touch screen</u>** (e.g. smart phone such as iPhone, Samsung Galaxy)	○	○	○	○	○	○
**<u>Handheld</u> electronic games** (e.g. PSP, Nintendo DS)	○	○	○	○	○	○
**<u>NON-ACTIVE</u> game consoles (playing sitting, not actively moving)** (e.g. XBOX, Wii, PS3)	○	○	○	○	○	○

<u>**ACTIVE**</u> **game consoles (playing actively and moving about)** (e.g. XBOX Kinect, Wii, PS3 MOVE)	○	○	○	○	○	○

How much do you use technology on weekdays and weekend days?
Think about about how often and how long you usually use technology with a screen. Technology includes television, desktop and laptop computers, tablet computers, mobile phones, and electronic games.

	**WEEKDAY (Mon-Fri)** On each of these **weekdays**, for about **how long** do you use screen devices **per day?**	**WEEKDAY (Mon-Fri)** Over a typical **Monday to Friday,** on **how many days** do you use screen devices?	**WEEKEND (Sat-Sun)** Over a typical **weekend** (Saturday to Sunday), on **how many days** do you use screen devices?	**WEEKEND (Sat-Sun)** On each of these **weekend days**, for about **how long** do you use screen devices **per day?**
**ALL Screen Devices**	▼ Please select ... 12 hours or more	▼ Please select ... 5 days	▼ Please select ... 2 days	▼ Please select ... 12 hours or more

How much do you use these devices on weekdays and weekend days?

	**WEEKDAY (Mon-Fri)** On each of these weekdays, for about **how long** do you use this device **per day?**	**WEEKDAY (Mon-Fri)** Over a typical **Monday to Friday period,** on **how many days** do you use this device?	**WEEKEND (Sat-Sun)** Over a typical weekend **(Saturday to Sunday),** on **how many days** do you use this device?	**WEEKEND (Sat-Sun)** On each of these weekend day, for about **how long** do you use this device **per day?**

**Television**	▼ Please select ... 12 hours or more	▼ Please select ... 5 days	▼ Please select ... 2 days	▼ Please select ... 12 hours or more
**Desktop computer**	▼ Please select ... 12 hours or more	▼ Please select ... 5 days	▼ Please select ... 2 days	▼ Please select ... 12 hours or more
**Laptop computer**	▼ Please select ... 12 hours or more	▼ Please select ... 5 days	▼ Please select ... 2 days	▼ Please select ... 12 hours or more
**Tablet computer** (e.g. iPad, Samsung Galaxy Tab, Kindle e-reader, Microsoft Surface Pro)	▼ Please select ... 12 hours or more	▼ Please select ... 5 days	▼ Please select ... 2 days	▼ Please select ... 12 hours or more
**Mobile phone - WITH touch screen** (e.g. smart phone such as iPhone, Samsung Galaxy)	▼ Please select ... 12 hours or more	▼ Please select ... 5 days	▼ Please select ... 2 days	▼ Please select ... 12 hours or more
**Handheld electronic games** (e.g. PSP, Nintendo DS)	▼ Please select ... 12 hours or more	▼ Please select ... 5 days	▼ Please select ... 2 days	▼ Please select ... 12 hours or more
**NON-ACTIVE game consoles (playing sitting, not actively moving)** (e.g. XBOX, Wii, PS3)	▼ Please select ... 12 hours or more	▼ Please select ... 5 days	▼ Please select ... 2 days	▼ Please select ... 12 hours or more
**ACTIVE game consoles (playing actively and moving about)** (e.g. XBOX Kinect, Wii, PS3 MOVE)	▼ Please select ... 12 hours or more	▼ Please select ... 5 days	▼ Please select ... 2 days	▼ Please select ... 12 hours or more

How much do you use more than one device at the same time - "Multi-tasking"?

	How often do you **multitask when you are using the following devices** (that is use another electronic device to do other activities at the same time, such as texting messages, play games, or watch videos)
**Television**	▼ Please select ... I never multitask
**Desktop computer**	▼ Please select ... I never multitask
**Laptop computer**	▼ Please select ... I never multitask
**Tablet computer** (e.g. iPad, Samsung Galaxy Tab, Kindle e-reader)	▼ Please select ... I never multitask
**Mobile phone - WITH touch screen** (e.g. smart phone such as iPhone, Samsung Galaxy)	▼ Please select ... I never multitask
**Handheld electronic games** (e.g. PSP, Nintendo DS)	▼ Please select ... I never multitask
**NON-ACTIVE game consoles (playing sitting, not actively moving)** (e.g. XBOX, Wii, PS3)	▼ Please select ... I never multitask
**ACTIVE game consoles (playing actively and moving about)** (e.g. XBOX Kinect, Wii, PS3 MOVE)	▼ Please select ... I never multitask

Page Break

The next 4 pages will ask you about what you use these devices for...

What do you use a <u>DESKTOP COMPUTER</u> for on weekdays and weekends?

	**WEEKEND (Sat-Sun)** Thinking about a typical **<u>Saturday to Sunday</u>,** about how long do you **<u>spend doing the following activities on desktop computer each day?</u>**	**WEEKDAY (Mon-Fri)** Thinking about a typical **<u>Monday to Friday</u>,** about how long do you **<u>spend doing the following activities on desktop computer each day</u>**?
**Doing school work in school, and homework at home** (e.g. using Microsoft Word, searching information online)	▼ Please select ... 12 hours or more	▼ Please select ... 12 hours or more
**Social networking/ instant messenger** (e.g. Facebook, Instagram, Twitter, WhatsApp, Snapchat, Skype, texting, voice calls)	▼ Please select ... 12 hours or more	▼ Please select ... 12 hours or more
**Watching videos/ shows** (e.g. watching movies, dramas, YouTube)	▼ Please select ... 12 hours or more	▼ Please select ... 12 hours or more
**Playing games**	▼ Please select ... 12 hours or more	▼ Please select ... 12 hours or more
**Other general use, <u>NOT</u> for school work** (e.g. visiting websites, Google, online shopping, downloading music, sending/receiving emails)	▼ Please select ... 12 hours or more	▼ Please select ... 12 hours or more

What do you use a LAPTOP COMPUTER for on weekdays and weekends?

	**WEEKEND (Sat- Sun)** Thinking about a typical **Saturday to Sunday,** about how long do you **spend doing the following activities on laptop computer each day?**	**WEEKDAY (Mon – Fri)** Thinking about a typical **Monday to Friday,** about how long do you **spend doing the following activities on laptop computer each day**?
**Doing school work in school, and homework at home** (e.g. using Microsoft Word, searching information online)	▼ Please select ... 12 hours or more	▼ Please select ... 12 hours or more
**Social networking/ instant messenger** (e.g. Facebook, Instagram, Twitter, WhatsApp, Snapchat, Skype, texting, voice calls)	▼ Please select ... 12 hours or more	▼ Please select ... 12 hours or more
**Watching videos/ shows** (e.g. watching movies, dramas, YouTube)	▼ Please select ... 12 hours or more	▼ Please select ... 12 hours or more
**Playing games**	▼ Please select ... 12 hours or more	▼ Please select ... 12 hours or more
**Other general use, NOT for school work** (e.g. visiting websites, Google, online shopping, downloading music, sending/receiving emails)	▼ Please select ... 12 hours or more	▼ Please select ... 12 hours or more

What do you use a TABLET COMPUTER for on weekdays and weekends?

	**WEEKEND (Sat-Sun)** Thinking about a typical **Saturday to Sunday,** about how long do you **spend doing the following activities on tablet computer each day?**	**WEEKDAY (Mon-Fri)** Thinking about a typical **Monday to Friday,** about how long do you **spend doing the following activities on tablet computer each day**?
**Doing school work in school, and homework at home** (e.g. using Microsoft Word, searching information online)	▼ Please select ... 12 hours or more	▼ Please select ... 12 hours or more
**Social networking/ instant messenger** (e.g. Facebook, Instagram, Twitter, WhatsApp, Snapchat, Skype, texting, voice calls)	▼ Please select ... 12 hours or more	▼ Please select ... 12 hours or more
**Watching videos/ shows** (e.g. watching movies, dramas, YouTube)	▼ Please select ... 12 hours or more	▼ Please select ... 12 hours or more
**Playing games**	▼ Please select ... 12 hours or more	▼ Please select ... 12 hours or more
**Other general use, NOT for school work** (e.g. visiting websites, Google, online shopping, downloading music, sending/receiving emails)	▼ Please select ... 12 hours or more	▼ Please select ... 12 hours or more

What do you use a MOBILE PHONE for on weekdays and weekends?

	**WEEKEND(Sat-Sun)**Thinking about a typical**<u>Saturday to Sunday</u>,** about how long do you**<u>spend doing the following activities on mobile phone (with touch screen) each day?</u>**	**WEEKDAY (Mon-Fri)** Thinking about a typical **<u>Monday to Friday</u>,** about how long do you **<u>spend doing the following activities on mobile phone (with touch screen) each day</u>**?
**Doing school work in school, and homework at home** (e.g. using Microsoft Word, searching information online)	▼ Please select ... 12 hours or more	▼ Please select ... 12 hours or more
**Social networking/ instant messenger** (e.g. Facebook, Instagram, Twitter, WhatsApp, Snapchat, Skype, texting, voice calls)	▼ Please select ... 12 hours or more	▼ Please select ... 12 hours or more
**Watching videos/ shows** (e.g. watching movies, dramas, YouTube)	▼ Please select ... 12 hours or more	▼ Please select ... 12 hours or more
**Playing games**	▼ Please select ... 12 hours or more	▼ Please select ... 12 hours or more
**Other general use, <u>NOT</u> for school work** (e.g. visiting websites, Google, online shopping, downloading music, sending/receiving emails)	▼ Please select ... 12 hours or more	▼ Please select ... 12 hours or more

What other sitting activities do you do (NOT using technology)?

	**WEEKEND (Sat - Sun)** Thinking about a typical **<u>Saturday to Sunday</u>,** about how long do you spend **<u>each day sitting down doing</u>:**	**WEEKDAY (Mon – Fri)** Thinking about a typical **<u>Monday to Friday</u>,** about how long do you spend **<u>each day sitting down doing</u>:**
**Homework/ school work (at home & outside school)** (**<u>NOT</u>** on a computer or other devices)	▼ Please select ... 12 hours or more	▼ Please select ... 12 hours or more
**Reading books/ paper** (**<u>NOT</u>** on a computer or other devices)	▼ Please select ... 12 hours or more	▼ Please select ... 12 hours or more
**Playing/ practising any musical instrument(s)**	▼ Please select ... 12 hours or more	▼ Please select ... 12 hours or more
**Art and crafts/ hobbies**	▼ Please select ... 12 hours or more	▼ Please select ... 12 hours or more
**Other sitting down time, such as chatting with friends/ playing card or board games etc.** (**<u>NOT</u>** on any electronic devices)	▼ Please select ... 12 hours or more	▼ Please select ... 12 hours or more

Some questions about your physical activity...

Thinking about the last 7 days, <u>on how many days</u> did you do physical activity for at least 60 minutes that increased your heart rate so you were out of breath? The 60 minutes each day could be broken up in shorter bursts throughout the day.

- ○ No days
- ○ 1 day
- ○ 2 days
- ○ 3 days
- ○ 4 days
- ○ 5 days
- ○ 6 days
- ○ 7 days

Over the last year, did you participate in **<u>organised team sport</u>** (eg. basketball, soccer, netball, football)? This is where you would usually play 1 to 3 times per week for a school term or the entire sporting season.

- ○ Yes, I ave participated in organised team sport
- ○ No, I haven't participated in organised team sport

Over the last year, did you participate in **<u>organised individual sports</u>** or physical activity on a regular basis (e.g. martial arts, dance)? This is where you would usually attend 1 to 3 times per week for a school term or the entire sporting competition.

- ○ Yes, I have participated in an organised individual sport
- ○ No, I haven't participated in an organised individual sport

---

Over the past 7 days, on an **average each day** how much time did you spend in **'non-organised' physical activity (not in school time)**? For example, e.g. kicking a ball against the wall, doing household chores or running around with your dog at the park)?

▼ Don't do it ... 12 or more hours per day

Some questions about your usual sleep...
Most nights do you feel you get...

- ◯ Too much sleep?
- ◯ The right amount of sleep?
- ◯ Too little sleep?

Most nights, do you consider yourself to be...

- ◯ A great sleeper?
- ◯ A good sleeper?
- ◯ An okay sleeper?
- ◯ A poor sleeper?

On **weekdays** (during school terms), what time do you **usually** go to bed?

- ◯ Before 7:00 pm
- ◯ 7:00-7:29 pm
- ◯ 7:30-7:59 pm
- ◯ 8:00-8:29 pm
- ◯ 8:30-8:59 pm
- ◯ 9:00-9:29 pm
- ◯ 9:30-9:59 pm
- ◯ 10:00-10:29 pm
- ◯ 10:30-10:59 pm
- ◯ 11:00-11:29 pm
- ◯ 11:30-11:59 pm
- ◯ After 12:00 am

On **weekdays** (during school terms), what time do you **usually** get up?

- ○ Before 5:00 am
- ○ 5:00-5:29 am
- ○ 5:30-5:59 am
- ○ 6:00-6:29 am
- ○ 6:30-6:59 am
- ○ 7:00-7:29 am
- ○ 7:30-7:59 am
- ○ 8:00-8:29 am
- ○ After 8:30 am

On **weekends**, what time do you **usually** go to bed?

- Before 8:00 pm
- 8:00-8:29 pm
- 8:30-8:59 pm
- 9:00-9:29 pm
- 9:30-9:59 pm
- 10:00-10:29 pm
- 10:30-11:00 pm
- 11:30-11:59 pm
- 12:00-12:29 am
- 12:30-1:00 am
- After 1:00 am

On **weekends**, what time do you **usually** get up?

- ○ Before 6:00 am
- ○ 6:00-6:29 am
- ○ 6:30-6:59 am
- ○ 7:00-7:29 am
- ○ 7:30-7:59 am
- ○ 8:00-8:29 am
- ○ 8:30-8:59 am
- ○ 9:00-9:29 am
- ○ 9:30-9:59 am
- ○ 10:00-10:30 am
- ○ 10:30-10:59 am
- ○ After 11:00 am

Some questions about any discomfort, soreness, ache or pain that you may have...
*Please answer for every part of your body, **one ANSWER only** for every question.*

The shaded area in the picture shows a body part.

Questions on this page refer to your <u>NECK/ SHOULDERS</u>, area as shaded in the picture below.

---

3. Have you had any discomfort, soreness, ache or pain at any time on your neck/ shoulders during the **<u>last month</u>?**

- ○ No
- ○ Yes

---

4. **<u>How often</u>** have you had the discomfort, soreness, ache or pain on your neck/ shoulders during the **last month?**

- ○ Almost never (less than once a month)
- ○ Occasionally (1-3 times a month)
- ○ Often (1-3 times a week)
- ○ Always (More than 3 times a week)

---

5. On average, what number (0-10 scale) best describes **<u>the amount of discomfort, soreness, ache or pain</u>** that you have had on your neck/shoulders in the **last month**?

0   = No discomfort
10 = Worst possible discomfort

- ◯ 0
- ◯ 1
- ◯ 2
- ◯ 3
- ◯ 4
- ◯ 5
- ◯ 6
- ◯ 7
- ◯ 8
- ◯ 9
- ◯ 10

---

6. On average, what number (0-10 scale) best describes **<u>the amount of interference to your normal studies/activities (at home or away from home)</u>**, caused by the discomfort, soreness, ache or pain on your neck/ shoulders in the **last month**?

0 = No interference
10 = Most interference

- ○ 0
- ○ 1
- ○ 2
- ○ 3
- ○ 4
- ○ 5
- ○ 6
- ○ 7
- ○ 8
- ○ 9
- ○ 10

Some questions about any discomfort, soreness, ache or pain that you may have...

Questions on this page refer to your <u>LOW BACK,</u> area as shaded in the picture below.

---

3. Have you had any discomfort, soreness, ache or pain at any time on your low back during the **<u>last month</u>?**

- ◯ No
- ◯ Yes

---

4. **<u>How often</u>** have you had the discomfort, soreness, ache or pain on your low back during the last month?

- ◯ Almost never (less than once a month)
- ◯ Occasionally (1-3 times a month)
- ◯ Often (1-3 times a week)
- ◯ Always (More than 3 times a week)

---

5. On average, what number (0-10 scale) best describes **<u>the amount of discomfort, soreness, ache or pain</u>** that you have had on your low back in the **last month**?

0   = No discomfort
10 = Worst possible discomfort

- ○ 0
- ○ 1
- ○ 2
- ○ 3
- ○ 4
- ○ 5
- ○ 6
- ○ 7
- ○ 8
- ○ 9
- ○ 10

6. On average, what number (0-10 scale) best describes **the amount of interference to your normal studies/activities (at home or away from home)**, caused by the discomfort, soreness, ache or pain on your low back in the **last month**?

0   = No interference
10 = Most interference

- ○ 0
- ○ 1
- ○ 2
- ○ 3
- ○ 4
- ○ 5
- ○ 6
- ○ 7
- ○ 8
- ○ 9
- ○ 10

Some questions about any discomfort, soreness, ache or pain that you may have...

Questions on this page refer to your <u>ARMS (including elbows, wrist, fingers)</u>, area as shaded in the picture below.

3. Have you had any discomfort, soreness, ache or pain at any time on your arms during the **<u>last month</u>?**

○ No

○ Yes

4. **<u>How often</u>** have you had the discomfort, soreness, ache or pain on your arms during the last month?

○ Almost never (less than once a month)

○ Occasionally (1-3 times a month)

○ Often (1-3 times a week)

○ Always (More than 3 times a week)

5. On average, what number (0-10 scale) best describes **<u>the amount of discomfort, soreness, ache or pain</u>** that you have had on your arms in the **last month**?

0   = No discomfort
10 = Worst possible discomfort

- ◯ 0
- ◯ 1
- ◯ 2
- ◯ 3
- ◯ 4
- ◯ 5
- ◯ 6
- ◯ 7
- ◯ 8
- ◯ 9
- ◯ 10

---

6. On average, what number (0-10 scale) best describes **the amount of interference to your normal studies/activities (at home or away from home)**, caused by the discomfort, soreness, ache or pain on your arms in the **last month**?

0 = No interference
10 = Most interference

- ○ 0
- ○ 1
- ○ 2
- ○ 3
- ○ 4
- ○ 5
- ○ 6
- ○ 7
- ○ 8
- ○ 9
- ○ 10

Some questions about your eyes...

1. Have you experienced any of the following symptoms **during or after using desktop computers, laptop computers, tablets, or smart phones?** (You can tick more than one option which applies to you)

- ☐ Eye strain (irritation, heaviness)
- ☐ Tiredness of eyes
- ☐ Watering of eyes
- ☐ Redness of eyes
- ☐ Itching of eyes
- ☐ Blurring of vision
- ☐ Dry eye
- ☐ Double vision
- ☐ Headache
- ☐ No symptoms

---

2. How often do your eyes feel dry (not wet enough)?

- ○ Never
- ○ Sometimes
- ○ Often
- ○ Constantly

---

3. How often do your eyes feel irritated?

- ○ Never
- ○ Sometimes
- ○ Often
- ○ Constantly

---

4. Have you ever been diagnosed (by a doctor/health professional) as having dry eye syndrome?

- ○ Yes
- ○ No

---

5. Do you wear glasses or contact lens?

- ○ Yes
- ○ No

Some questions about your visual health...

6. If yes, when you are not wearing glasses/contact lens, do you have trouble with any of the following?

- ☐ Seeing things far away – short sightedness (myopia)
- ☐ Seeing things near – long sightedness (hyperopia)
- ☐ I don’t have trouble seeing things far away or near

What do you think about technology? (Part 1)

In this section we want to find out what YOUR opinion about technology is. By technology we mean things like computers, tablets, mobile phones, and the Internet. Please answer the questions and let us know what you think about technology. There are no right or wrong answers so just mark the number that comes closest to what YOU think. Please answer all items.

How much do you disagree or agree with the following statements:

	Strongly Agree	Agree	Disagree	Strongly Disagree
Technology is very important in life	○	○	○	○
Technology makes school more interesting	○	○	○	○
Working in technology is very creative	○	○	○	○
Girls can do technology as well as boys	○	○	○	○
I positively do NOT want to have a job that uses a lot of technology.	○	○	○	○
People make a lot of money using technology	○	○	○	○
Technology is too difficult for me	○	○	○	○
For students my age, technology is NOT interesting	○	○	○	○
I would like to learn more about technology at school	○	○	○	○
I am NOT interested in technology	○	○	○	○
At school you do NOT hear much about technology	○	○	○	○

I think technology is a little scary	○	○	○	○
Technology is as difficult for girls as it is for boys	○	○	○	○
Technology is only for bright people	○	○	○	○
Working with computers is boring	○	○	○	○

This is the last section!!!

What do you think about technology? (Part 2)

In this section we want to find out what YOUR opinion about technology is. By technology we mean things like computers, tablets, mobile phones, and the Internet. Please answer the questions and let us know what you think about technology. There are no right or wrong answers so just mark the number that comes closest to what YOU think. Please answer all items.

How much do you disagree or agree with the following statements:

	Strongly Agree	Agree	Disagree	Strongly Disagree
I will probably need to know how to use a computer when I leave school	○	○	○	○
I like using computers in my schoolwork	○	○	○	○
I feel comfortable working with computers	○	○	○	○
I like reading books better than computer screens	○	○	○	○
Video games are good for making me think	○	○	○	○
I like seeing videos in class	○	○	○	○
I learn more when teachers use videos and computers than when they don’t	○	○	○	○
Technology is unreliable and doesn’t usually work when you want it to	○	○	○	○
I’m relaxed when I work with a computer	○	○	○	○
I can do a good job using technology	○	○	○	○
I’m really used to using technology	○	○	○	○

I think I use technology too much	○	○	○	○
My parents think I should use technology less	○	○	○	○
I am happy with how much I use technology	○	○	○	○

You've finished!!!
